# Fatal obstructive uropathy secondary to neglected benign prostatic hyperplasia: A medico-legal case report

**DOI:** 10.1097/MD.0000000000047280

**Published:** 2026-01-16

**Authors:** Di Liang, RunWu Chen, YingJun Chen, HongYu Su, LiJie Su, XinYu Liang, Xian Ju

**Affiliations:** a School of Forensic Medicine, Shanxi Medical University, Jinzhong, Shanxi Province, China; b Department of Pathology, Nanning Zhongyi Forensic Science Institute, Nanning, Guangxi, China; c First Department of Surgery, Guangxi Zhuang Autonomous Region Institute for the Prevention and Treatment of Occupational Disease, Nanning, Guangxi, China; d West China School of Basic Medical Sciences and Forensic Medicine, Sichuan University, Chengdu, Sichuan Province, China.

**Keywords:** autopsy, benign prostatic hyperplasia, case report, forensic pathology, obstructive nephropathy, renal failure, urinary retention

## Abstract

**Rationale:**

Benign prostatic hyperplasia (BPH) is a common, typically nonlethal condition in middle-aged and older men. However, its complications can lead to fatal outcomes if untreated. This report presents a rare fatal case of obstructive uropathy and renal failure resulting from neglected BPH.

**Patient concerns:**

A 54-year-old reclusive male with no known medical interventions was found deceased at home after being unresponsive to contact for several days.

**Diagnoses:**

Postmortem examination revealed marked bladder distension (2500 mL), hemorrhagic cystitis, prostatic enlargement (45 g) with urethral obstruction, bilateral hydronephrosis, and renal cortical cysts. Histopathology confirmed obstructive nephropathy and renal failure. Extreme elevations in postmortem serum creatinine (879 μmol/L) and urea (153.4 mmol/L) supported the diagnosis of terminal uremia. Toxicological screening was negative.

**Interventions:**

No medical interventions were undertaken prior to death.

**Outcomes:**

The patient died due to complications of BPH, specifically urinary retention leading to obstructive nephropathy, renal failure (uremia), and subsequent multi-organ dysfunction syndrome.

**Lessons:**

This case underscores the potentially lethal consequences of untreated bladder outlet obstruction from BPH. It highlights the importance of clinical vigilance, early intervention, and patient education, particularly for at-risk individuals such as those living alone or with limited healthcare access.

## 1. Introduction

Benign prostatic hyperplasia (BPH) is a prevalent condition among middle-aged and elderly males, frequently associated with lower urinary tract symptoms. Although not inherently fatal, complications of BPH (e.g., urinary retention, hydronephrosis, postrenal failure) can threaten health and life.^[1–3]^ Deaths attributable to genitourinary causes are relatively uncommon in forensic practice, with literature primarily documenting cases linked to trauma, infection, malignancy, or iatrogenic complications.^[4–6]^ However, forensic reports detailing death solely from the uncomplicated, natural progression of BPH to obstructive nephropathy and uremia are exceptionally rare in the modern medical literature, making this case a pertinent educational example.

## 2. Case presentation

The deceased was a 54-year-old male who lived reclusively. According to family members, repeated attempts to contact him by phone over several days yielded no response. He was subsequently discovered deceased within his rented residence. Law enforcement officers arrived at the scene and conducted a preliminary investigation. External examination revealed minor abrasions on the body surface. However, no signs of physical struggle or environmental disturbance were observed. Forensic analysis at the scene revealed no suspicious findings, leading investigators to rule out homicide as a potential cause-of-death.

**Figure 1.**
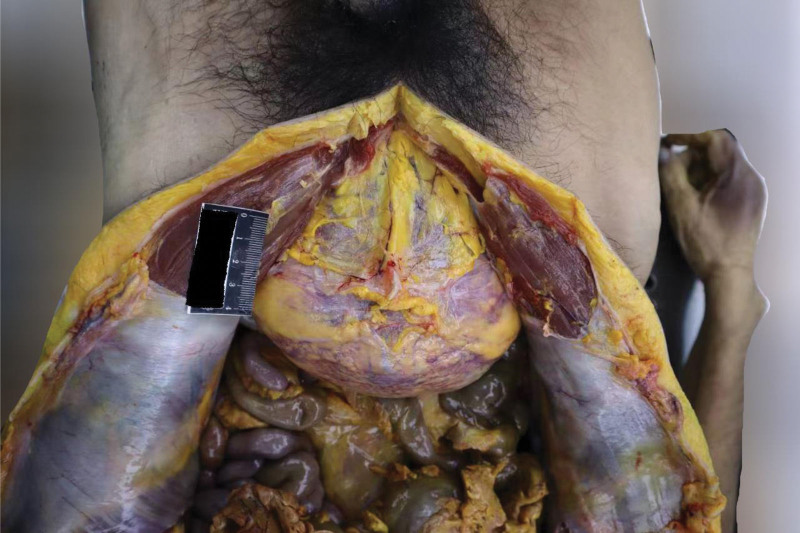
The gross photograph shows massively distended urinary bladder.

**Figure 2.**
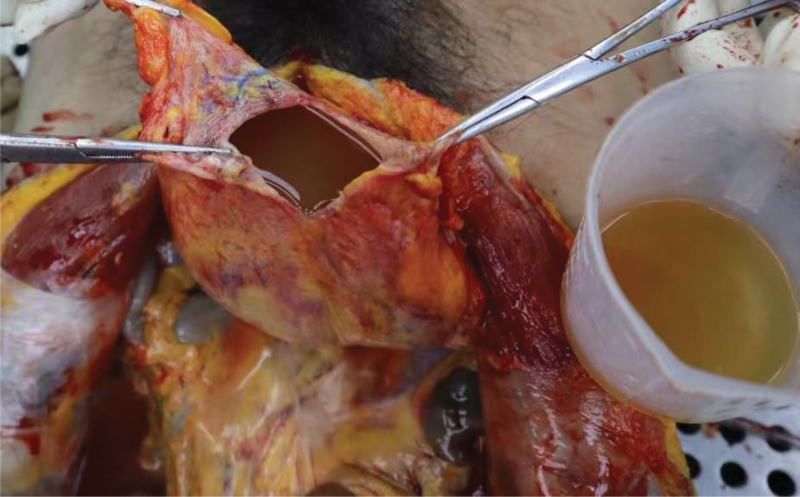
Hemorrhagic fluid within the opened bladder (volume: 2500 mL).

**Figure 3.**
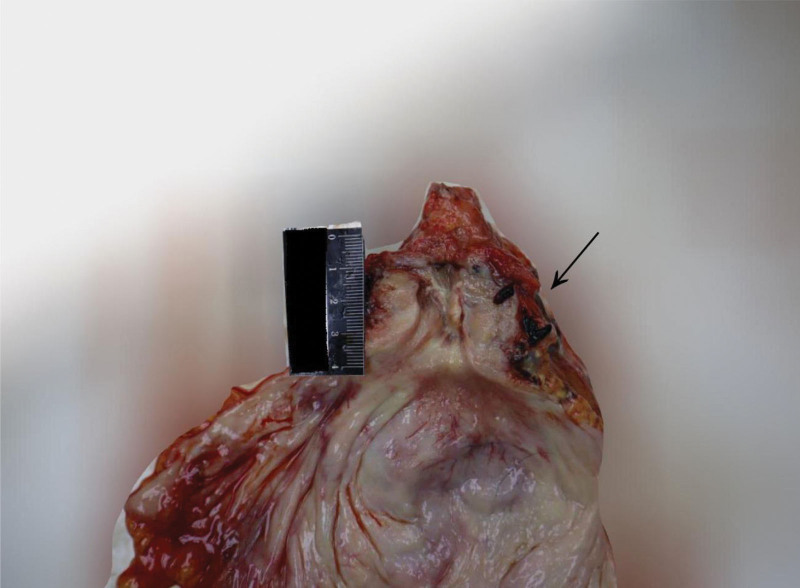
Prostatic enlargement (45 g) with urethral stenosis (arrow).

**Figure 4.**
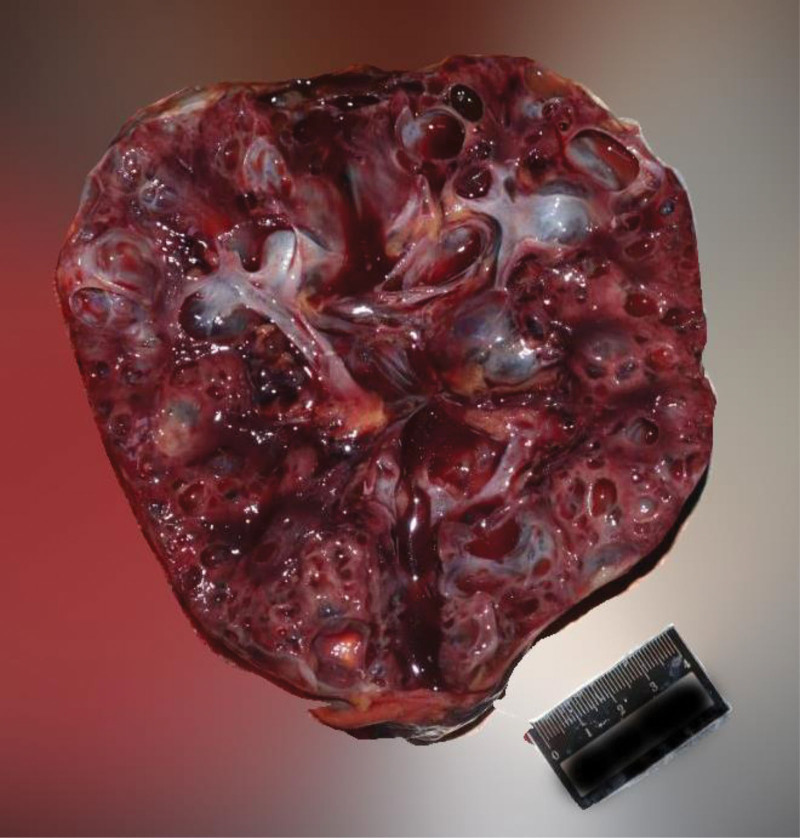
Bilateral multiple renal cortical cysts.

**Figure 5.**
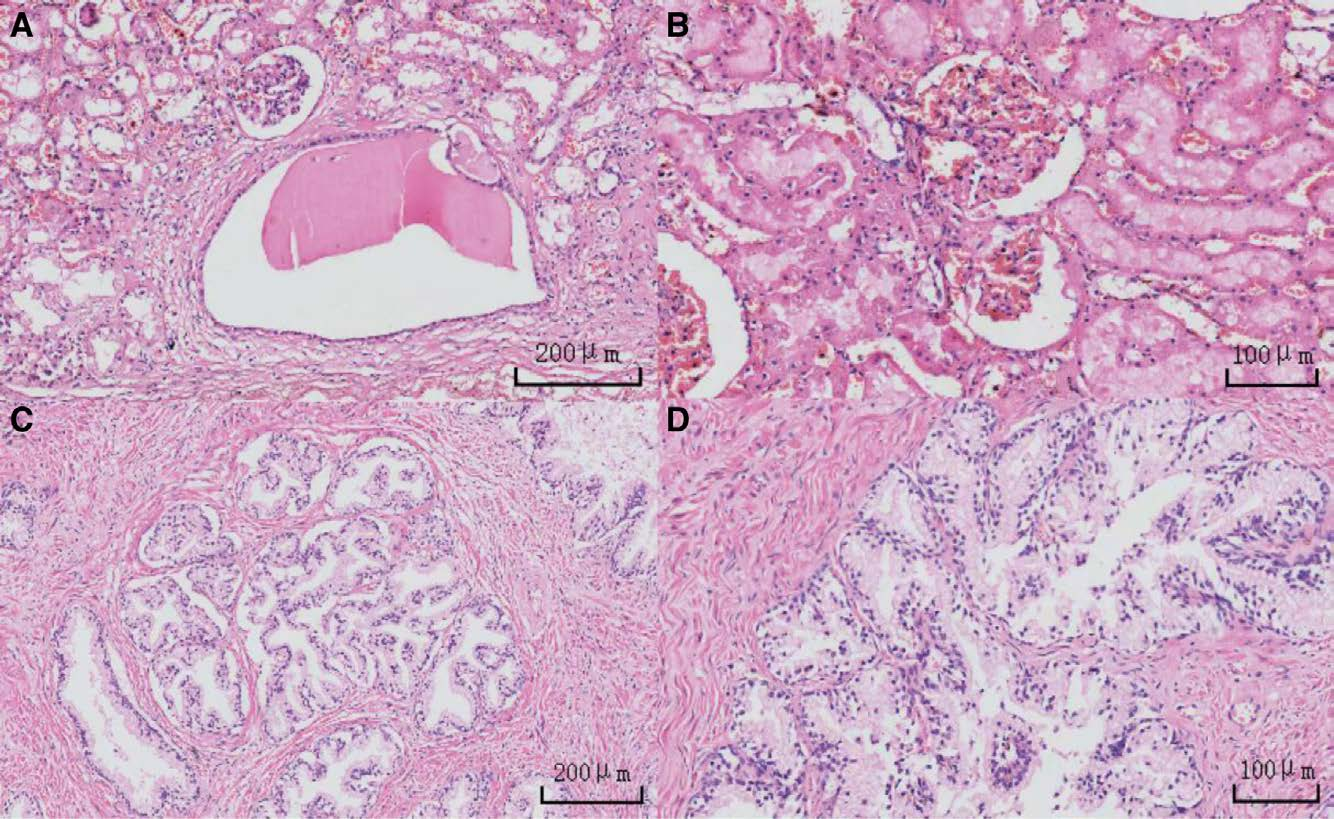
Histopathological findings (H&E staining). (A and B) Congestion of red blood cells within glomerular capillary loops, hydropic degeneration of proximal convoluted tubules, congestion of interstitial small vessels (original magnification: ×100 for A; ×200 for B). (C and D) Cystic hyperplastic acini of prostate (original magnification: ×100 for C; ×200 for D).

The autopsy was conducted 5 days after the estimated time of death, based on postmortem changes including resolved rigor mortis and fixed lividity. External examination of the body showed dark red lividity distributed over the neck, back, hypochondrium, and the unpressed dorsal aspects of the limbs. Rigor mortis had completely resolved. No mechanical injuries were observed elsewhere. Internal examination revealed a distended and enlarged urinary bladder (Fig. 1) containing 2500 mL of pale red fluid (Fig. 2), with hemorrhage in the bladder mucosa. The prostate gland weighed 45 g (the normal adult prostate weight ranges from 15–30 g) and measured 4.8 × 3.8 × 3.5 cm, with stenosis observed at the posterior urethral orifice (Fig. 3). Bilateral multiple renal cortical cysts were observed (Fig. 4). The left kidney weighed 200 g and measured 10 × 16 × 6 cm; the right kidney weighed 176 g and measured 8 × 14 × 7 cm. No significant abnormalities were noted in the remaining organs. Histopathological examination revealed the following microscopic findings: Kidneys: Congestion of red blood cells within glomerular capillary loops, hydropic degeneration of proximal convoluted tubules, congestion of interstitial small vessels, and renal cysts (Fig. 5A and B). Bladder: Neutrophil infiltration extending from the mucosa into the muscularis and serosal layers, with patchy red blood cell infiltration within the muscularis and serosa. Prostate: Cystic hyperplastic acini (Fig. 5C and D). Brain, liver, spleen, and pancreas: Congestion and edema. No lethal mechanical injuries or pathological changes indicative of fatal disease were identified in the remaining organs. Postmortem biochemical analysis of cardiac blood yielded the following results: Creatinine, 879 μmol/L (reference range: 59–104 μmol/L). Urea, 153.4 mmol/L (reference range: 2.9–7.5 mmol/L).

Scene examination identified no medications or medical devices. Toxicological analysis of heart blood and gastric contents by gas chromatography-mass spectrometry (GC-MS) yielded no detectable levels of common drugs of abuse, ethanol, or over-the-counter analgesic agents above established therapeutic or toxic thresholds.

Based on the integration of the available evidence, we conclude that the cause-of-death was complications of BPH, specifically urinary retention leading to obstructive nephropathy, renal failure (uremia), and multi-organ dysfunction syndrome.

## 3. Discussion

BPH typically causes lower urinary tract symptoms classified as irritative, obstructive, or mixed.^[1]^ Although not inherently lethal, untreated BPH may progress to fatal complications such as urinary retention, obstructive nephropathy, and postrenal failure.^[3]^ In this case, prostatic enlargement induced complete urethral obstruction, triggering acute urinary retention. The resulting massive bladder distension (2500 mL) significantly exceeded critical capacity thresholds (>1000 mL indicates severe retention), which directly explains the observed hemorrhagic cystitis and bilateral hydronephrosis. Histopathological evidence (glomerular congestion, tubular hydropic degeneration, renal cysts) and extreme biochemical derangements (creatinine: 879 μmol/L; urea: 153.4 mmol/L) confirmed terminal uremia. Multi-organ congestion and edema signified end-stage systemic failure secondary to renal dysfunction.

In many cases, lower urinary tract obstruction due to BPH can progress insidiously. Patients may remain asymptomatic or attribute mild symptoms to aging, leading to delayed medical consultation. Chronic bladder overflow and bilateral hydroureteronephrosis can develop without overt symptoms until late stages, when signs such as nocturnal incontinence or uremic symptoms (e.g., fatigue, nausea) emerge. Remarkably, some patients present with extremely high creatinine levels (e.g., >20 mg/dL) yet remain conscious, underscoring the variability in renal compensatory mechanisms. In this case, the deceased reclusive lifestyle and lack of medical intervention allowed for unchecked progression to fatal renal failure.

The causal pathway from BPH to death in this case is supported by a combination of anatomic, histopathologic, and biochemical findings. Microbiological culture of bladder fluid was not performed due to advanced autolysis. However, the absence of pyelonephritis on histopathology and systemic signs of sepsis (e.g., splenic hyperemia was reactive, not infective) supports that infection was not the primary cause-of-death. Similarly, while bladder distension could theoretically contribute to abdominal compartment syndrome,^[7–9]^ the chronic clinical course and lack of acute abdominal signs make abdominal compartment syndrome unlikely. Hyperkalemia-induced arrhythmia, a common terminal event in renal failure, may have contributed but is considered a consequence rather than an independent cause. Postmortem biochemical values can be influenced by factors such as autolysis and redistribution. However, studies support the reliability of postmortem biochemistry even after some delay.^[10]^ For instance, Palmiere et al demonstrated that analysis of serum samples stored at–20°C remains valuable for cause-of-death determination.^[11]^ Therefore, despite the 5-day postmortem interval, the profound elevations in creatinine and urea, in conjunction with the definitive anatomic evidence of obstruction, provide robust support for the diagnosis of fatal uremia. Toxicologic screening ruled out exogenous toxins or drugs as contributory factors. Although the deceased presented with multiple renal cysts, these were predominantly localized simple cysts that did not occupy a significant portion of the renal parenchyma and were insufficient to solely account for end-stage renal failure. Furthermore, clear evidence of mechanical obstruction was identified, including marked prostatic hyperplasia and bilateral dilation of the renal pelvises and ureters. Therefore, we consider the renal cysts to be an incidental finding that may have reduced renal functional reserve, but the underlying cause of his renal failure (uremia) and eventual death was urinary tract obstruction secondary to BPH and its ensuing pathophysiological sequelae.

Recent advances in the management of prostate diseases, including both benign and malignant conditions, have expanded therapeutic options. Minimally invasive surgical techniques, novel medical therapies, and improved diagnostic tools facilitate early intervention and reduce complication rates.^[12–16]^ However, these advancements are ineffective if patients do not seek care. This case highlights the critical need for public health initiatives aimed at identifying at-risk individuals, particularly those living alone or with limited healthcare access, and educating them on the serious consequences of untreated urinary obstruction.

## 4. Conclusion

This rare case of fatal obstructive uropathy and uremia, resulting solely from neglected BPH, clearly demonstrates the potentially lethal consequences of untreated bladder outlet obstruction. It serves as a critical reminder to clinicians that BPH, while common and often manageable, can progress to catastrophic renal failure if left unaddressed. Vigilance in identifying at-risk patients (e.g., those living alone, dismissing symptoms, or with limited healthcare access), coupled with prompt intervention and thorough patient education on the dangers of untreated obstruction, is paramount to preventing such tragic outcomes.

## Acknowledgments

Thanks to the family of the deceased for agreeing to release this case report.

## Author contributions

**Conceptualization:** Di Liang, RunWu Chen, HongYu Su, LiJie Su, XinYu Liang, Xian Ju.

**Formal analysis:** Di Liang, LiJie Su, XinYu Liang, Xian Ju.

**Investigation:** RunWu Chen, XinYu Liang.

**Resources:** RunWu Chen, LiJie Su, XinYu Liang.

**Supervision:** Di Liang, HongYu Su, Xian Ju.

**Writing – original draft:** Di Liang, YingJun Chen, Xian Ju.

**Writing – review & editing:** Di Liang, RunWu Chen, YingJun Chen, HongYu Su, Xian Ju.
